# Increasing nonalcoholic fatty liver disease–related mortality rates in the United States from 1999 to 2022

**DOI:** 10.1097/HC9.0000000000000207

**Published:** 2023-07-03

**Authors:** Fariha Ilyas, Hassam Ali, Pratik Patel, Shiza Sarfraz, Debargha Basuli, Alexa Giammarino, Sanjaya Kumar Satapathy

**Affiliations:** 1 Department of Internal Medicine, ECU health medical center/Brody School of Medicine, Greenville, North Carolina, USA; 2 Department of Gastroenterology, Mather Hospital/Hofstra University Zucker School of Medicine, Port Jefferson, New York, USA; 3 Department of Internal Medicine, Quaid-e-Azam Medical College, Punjab, Pakistan; 4 Department of Internal Medicine, North Shore University Hospital/Zucker School of Medicine at Hofstra University, Manhasset, New York, USA; 5 Department of Hepatology, North Shore University Hospital/Zucker School of Medicine at Hofstra University, Manhasset, New York, USA

## Abstract

We examined trends in NAFLD-related mortality in the United States from 1999 to 2022, focusing on sex, racial differences, and specific age groups.

**Methods:**

We analyzed age-adjusted mortality rates (AAMRs) for NAFLD-related deaths using the Centers for Disease Control and Prevention Wide-Ranging Online Data for Epidemiologic Research database and assessed differences between sex and racial groups.

**Results:**

Between 1999 and 2022, NAFLD-related mortality rose from an age-adjusted mortality rate (AAMR) of 0.2 to 1.7 per 100,000, with an average annual percent change (AAPC) of 10.0% (*p* < 0.001). In all, 85.4% of the cases were reported after 2008. Females (0.2–2 per 100,000, AAPC: 11.7%, *p* < 0.001) saw a steeper increase than males (0.2–1.3 per 100,000, AAPC: 9.3%, *p* < 0.001). White individuals’ AAMR rose from 0.2 to 1.9 per 100,000 (AAPC: 10.8%, *p* < 0.001). Asian or Pacific Islanders (AAPI) increased from 0.2 in 2013 to 0.5 in 2022 (AAPC: 12.13%, *p* = 0.002), and American Indians or Alaska Natives (AI/AN) from 1 in 2013 to 2.2 in 2022 (AAPC: 7.9%, *p* = 0.001). African Americans (AA) showed an insignificant change (0.3–0.5 per 100,000, AAPC: 0.7%, *p* = 0.498). Regarding age, individuals 45–64 saw AAMR rise from 0.3 to 1.2 per 100,000 (AAPC: 6.5%, *p* < 0.001), and those 65+ from 0.2 to 6 per 100,000 (AAPC: 16.5%, *p* < 0.001). No change was observed in the 25–44 age group (AAMR: 0.2 per 100,000, AAPC: 0.0%, *p* = 0.008).

**Conclusion:**

We report increased NAFLD-related mortality among both sexes and certain racial groups. The mortality rate increased for older populations, emphasizing the need for targeted public health measures and evidence-based interventions.

## INTRODUCTION

NAFLD represents a significant public health concern, affecting a growing number of adults worldwide.^1^ The most common etiology of mortality in patients with chronic liver disease is NAFLD.^2^ Moreover, in the past few decades, a dramatic change in lifestyle behaviors resulting in an epidemic of obesity has led to an unprecedented increase in prevalence and mortality related to NAFLD.^3^ Given the increasing trends in obesity, NAFLD is becoming the leading indicator of liver transplantation, replacing Hepatitis C.^4^ According to epidemiologic studies, ethnicity plays a very important role in complications and treatment responses in patients with NAFLD. However, data on ethnic and racial disparities are very scarce.^5^ However, emergent evidence suggests the rising mortality rate in women compared to men, putting them at increased risk for negative outcomes.^6^ Given the evolving landscape of liver-related health issues, our study aims to investigate trends in NAFLD-related mortality in the United States over the past 2 decades.

## METHODS

This study followed the STROBE reporting guideline and did not require informed consent or institutional review board approval as it utilized publicly available data that were deidentified in compliance with the Common Rule.

We retrieved deidentified data from the Centers for Disease Control and Prevention Wide-Ranging Online Data for Epidemiologic Research multiple causes of death database (years 1999–2022) for NAFLD-related mortality with a focus on the underlying cause of death.^7^ We considered individuals with NAFLD defined by the International Classification of Diseases-10 codes (K75.8 or K76.0).^8,9^

We examined age-adjusted mortality rates (AAMRs) per 100,000 population to analyze cancer-related mortality rates from ages 25 years and above. AAMRs were standardized to the 2000 US population. Joinpoint software assessed temporal trends in the average percent change (APC), representing the change in mortality during a specific period. Joinpoint identified time points where the trend changed significantly using a Monte Carlo permutation test and a t-test. Log-linear regression models were fitted to evaluate trends in AAMRs. We fitted the simplest model by applying the smallest trend segments or periods, assuming a Poisson distribution and a maximum of 3 joinpoints. This method minimized bias when assessing trends in incidence or mortality rates. The final models estimated the APC, average annual percent change (AAPC), 95% CI, and *p* values. We conducted a nonparallel pairwise comparison using Joinpoint regression analysis to assess differences in trends between sex, race, and age group over time, reporting AAPC differences with 95% CI and *p* values. Statistical significance was established at *p* ≤ 0.05. Racial/ethnic groups were divided into Whites, African Americans (AA), Hispanics, Asian or Pacific Islanders (AAPI), and American Indians or Alaska Natives (AI/AN).

## RESULTS

**FIGURE 1:**
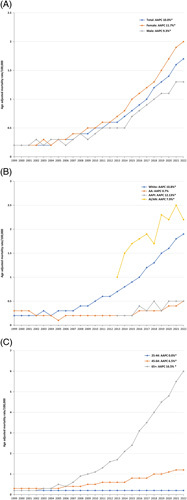
Trends in NAFLD-related mortality from 1999 to 2022 in the United States, stratified by sex, race, and age groups.

Between 1999 and 2022, 38,482 deaths related to NAFLD increased from 0.2/100,000 in 1999 to 1.7/100,000 in 2022, showing a significant average annual percentage change (AAPC) of 10.0% (95% CI: 8.8%–11.2%, *p* < 0.001) (Figure. 1A) (Supplemental Table 1, http://links.lww.com/HC9/A388). In all, 14.6% of the cases were reported before 2008 (Table 1). Overall, 98.9% of cases were coded as “K76.0,” while 1.1% were coded as “K75.8” (Supplemental Table 2, http://links.lww.com/HC9/A388). From 1999 to 2003, there was a nonsignificant increase in NAFLD-related mortality with an annual percentage change (APC) of 1.0% (95% CI: −5.0%–7.4%, *p* = 0.733). However, from 2003 to 2022, there was a significant increase in NAFLD-related mortality with an APC of 12.0% (95% CI: 11.4%–12.7%, *p* < 0.001).

**TABLE 1:** Age-adjusted rates of NAFLD-related mortality stratified by decedent sex, race, and age (1999–2020).

	Total deaths (N)	Age-adjusted mortality rate (AAMR)/100,000 for the study period	Average annual percent change (AAPC) for the study period	*p*
Total^a^	38,482	0.65	10% (95% CI: 8.8%–11.2%)	<0.001
1999–2007	4,161	0.23	6.3% (95% CI: 2.3%–10.4%)	0.007
2008–2022	34,321	0.64	11.6% (95% CI: 11%–12.3%)	<0.001
Sex				
Male	14817	0.66	9.3% (95% CI: 8.1%–10.4%	<0.001
Female	23665	0.75	11.7% (95% CI: 10.9%–12.5%)	<0.001
Race^b^				
White	35704	0.46	10.8% (95% CI: 9.4%–12.2%)	<0.001
African Americans (AA)	1532	0.2	0.7% (95% CI: −1.4%–2.9%)	0.498
Asian or Pacific Islanders (AAPI)	595	0.3	12.13%, 95% CI: 5.8%–18.8%	0.002
American Indians or Alaska Natives (AI/AN)	607	1.88	7.9%, 95% CI: 4.2%–11.8%)	0.001
Age groups				
25–44	3387	0.2	0.0% (95% CI: 0.0%–0.0%)	0.008
45–64	12550	0.4	6.5% (95% CI: 5.5%–7.6%)	<0.001
65+	22845	1.9	16.5% (95% CI: 13.5%–19.7%)	<0.001

a Query date: April 30, 2023, 1:19:16 PM.

b 44 deaths of unknown race and not included in the study for racial disparity.

For females, AAMR increased from 0.2/100,000 in 1999 to 2/100,000 in 2022, with an AAPC of 11.7% (95% CI: 10.9%–12.5%, *p* < 0.001). For males, AAMR increased from 0.2/100,000 in 1999 to 1.3/100,000 in 2022, with an AAPC of 9.3% (95% CI: 8.1%–10.4%, *p* < 0.001). The difference in AAPC between males and females was 2.4% (95% CI: 1.1%–3.7%, *p* < 0.001) (Figure. 1A).

For the AA race, AAMR increased from 0.3/100,000 in 1999 to 0.5/100,000 in 2022, showing an AAPC of 0.7% (95% CI: −1.4%–2.9%, *p* = 0.498). For the white race, AAMR increased from 0.2/100,000 in 1999 to 1.9/100,000 in 2022, showing an AAPC of 10.8% (95% CI: 9.4%–12.2%, *p* < 0.001). The difference in AAPC between White and AA populations was -10.1% (95% CI: −12.6% to –7.5%, *p* < 0.001). For AAPI, AAMR increased from 0.2/100,000 in 2013 to 0.5/100,000 in 2022 with a significant AAPC of 12.13% (95% CI: 5.8%–18.8%, *p* = 0.002). For AI/AN, AAMR increased from 1/100,000 in 2013 to 2.2/100,000 in 2022, with an AAPC of 7.9% (95% CI: 4.2%–11.8%, *p* = 0.001). Earlier years were not included in Joinpoint regression due to unreliable AAMRs for certain years (Figure. 1B).

For the age group 25–34 years, AAMR remained the same at 0.2/100,000 with an AAPC of 0.0% (95% CI: 0.0%–0.0%, *p* = 0.008). For individuals aged 45–64, AAMR increased from 0.3/100,000 in 1999 to 1.2/100,000 in 2022, with an AAPC of 6.5% (95% CI: 5.5%–7.6%, *p* < 0.001). Similarly, for individuals aged 65 and above, AAMR increased from 0.2/100,000 in 1999 to 6/100,000 in 2022, with an AAPC of 16.5% (95% CI: 13.5%–19.7%, *p* < 0.001). When comparing age groups, the difference in AAPC between the 45–64 age group and the 25–44 age group was −6.5% (95% CI: −7.6% to–5.5%, *p* < 0.001). Similarly, the difference in AAPC between the 65+ and 25–44 age groups was −16.5% (95% CI: −19.6% to–13.4%, *p* < 0.001) (Figure. 1C).

## DISCUSSION

Our investigation analyzed NAFLD-related mortality trends between 1999 and 2022, uncovering a notable escalation in mortality rates, particularly from 2003 to 2022. This upward trend was more prominent among females compared to males. Racial disparities were also evident, with White, AAPI, and AI/AN populations experiencing a rising trend, while AA individuals showed a nonsignificant change in AAPC. Most cases (85.4%) were reported from the year 2008 onward. Only 1.1% of the cases were coded as “K75.8,” which indicates “other specified inflammatory liver diseases.” Most cases were coded as “fatty (change of) liver, not elsewhere classified” (K76.0).

Age-specific analysis revealed that the increase in NAFLD-related mortality was more pronounced among the 45–64 and 65+ age groups, whereas no significant change was observed for individuals aged 25–44. The disparities between age groups emphasize the importance of targeted public health measures for older populations.

Our study is limited by potential misclassification bias in death certificate data; in addition, years 2021 and 2022 were extracted from the provisional multiple causes of death database, and the numbers may be subject to change when the dataset is finalized. However, it sheds light on the growing burden of NAFLD-related mortality and the disparities among sex, racial groups, and age brackets. As a result, researchers, policymakers, and health care professionals need to monitor these trends closely and develop evidence-based interventions to improve public health outcomes.

## Supplementary Material
