# Supplementary material for: Increasing nonalcoholic fatty liver disease–related mortality rates in the United States from 1999 to 2022
Source: Hepatol Commun. 2023 Jul 3;7(7):e00207. doi: 10.1097/HC9.0000000000000207 (PMC10319370; doi:10.1097/HC9.0000000000000207)
Supplement: Supplementary file 1 [file hc9-7-e00207-s001.docx]

**Supplementary Table 1** Age-adjusted nonalcoholic fatty liver disease–Related Mortality from 1999 to 2022 in the United States per 100 000 population.

| Year | Total: AAPC 10.0%* | Female: AAPC 11.7%* | Male: AAPC 9.3%* | White: AAPC 10.8%* | AA: AAPC 0.7% | AAPI: AAPC 12.13%* | AI/AN: AAPC 7.9%* | 25-44: AAPC 0.0%* | 45-64: AAPC 6.5%* | 65+: AAPC 16.5% * |
| --- | --- | --- | --- | --- | --- | --- | --- | --- | --- | --- |
| 1999 | 0.2 | 0.2 | 0.2 | 0.2 | 0.3 |  |  | 0.2 | 0.3 | 0.2 |
| 2000 | 0.2 | 0.2 | 0.2 | 0.2 | 0.3 |  |  | 0.2 | 0.3 | 0.2 |
| 2001 | 0.2 | 0.2 | 0.2 | 0.2 | 0.3 |  |  | 0.2 | 0.3 | 0.2 |
| 2002 | 0.2 | 0.2 | 0.3 | 0.2 | 0.2 |  |  | 0.2 | 0.3 | 0.2 |
| 2003 | 0.2 | 0.3 | 0.2 | 0.3 | 0.2 |  |  | 0.2 | 0.3 | 0.3 |
| 2004 | 0.2 | 0.2 | 0.3 | 0.2 | 0.2 |  |  | 0.2 | 0.3 | 0.3 |
| 2005 | 0.3 | 0.3 | 0.3 | 0.3 | 0.1 |  |  | 0.2 | 0.3 | 0.5 |
| 2006 | 0.3 | 0.3 | 0.2 | 0.3 | 0.2 |  |  | 0.2 | 0.4 | 0.4 |
| 2007 | 0.3 | 0.4 | 0.3 | 0.3 | 0.2 |  |  | 0.2 | 0.4 | 0.6 |
| 2008 | 0.4 | 0.4 | 0.4 | 0.4 | 0.2 |  |  | 0.2 | 0.4 | 0.9 |
| 2009 | 0.4 | 0.5 | 0.3 | 0.4 | 0.2 |  |  | 0.2 | 0.5 | 1 |
| 2010 | 0.5 | 0.5 | 0.4 | 0.5 | 0.2 |  |  | 0.2 | 0.5 | 1.1 |
| 2011 | 0.5 | 0.6 | 0.5 | 0.6 | 0.2 |  |  | 0.2 | 0.6 | 1.3 |
| 2012 | 0.6 | 0.6 | 0.5 | 0.6 | 0.2 |  |  | 0.2 | 0.6 | 1.6 |
| 2013 | 0.6 | 0.7 | 0.5 | 0.7 | 0.2 | 0.2 | 1 | 0.2 | 0.6 | 1.7 |
| 2014 | 0.7 | 0.8 | 0.5 | 0.8 | 0.2 | 0.2 | 1.5 | 0.2 | 0.6 | 2.1 |
| 2015 | 0.8 | 1 | 0.7 | 0.9 | 0.2 | 0.2 | 1.7 | 0.2 | 0.8 | 2.4 |
| 2016 | 0.9 | 1.1 | 0.8 | 1 | 0.3 | 0.4 | 1.8 | 0.2 | 0.8 | 3.1 |
| 2017 | 1 | 1.2 | 0.9 | 1.2 | 0.3 | 0.3 | 1.9 | 0.2 | 0.8 | 3.5 |
| 2018 | 1.2 | 1.3 | 1 | 1.3 | 0.3 | 0.5 | 1.7 | 0.2 | 1 | 4 |
| 2019 | 1.3 | 1.5 | 1.1 | 1.5 | 0.3 | 0.3 | 2.3 | 0.2 | 1 | 4.5 |
| 2020 | 1.4 | 1.7 | 1.1 | 1.6 | 0.4 | 0.5 | 2.2 | 0.2 | 1.1 | 4.8 |
| 2021 | 1.6 | 1.9 | 1.3 | 1.8 | 0.4 | 0.5 | 2.5 | 0.2 | 1.2 | 5.5 |
| 2022 | 1.7 | 2 | 1.3 | 1.9 | 0.5 | 0.5 | 2.2 | 0.2 | 1.2 | 6 |

AAPC: Annual Average Percent Change

*: Statistically significant

AA: African Americans

AAPI: Asian Americans and Pacific Islanders

AI/AN: American Indians/Alaska Natives

**Supplementary Table 2** Age-adjusted nonalcoholic fatty liver disease–Related Mortality from 1999 to 2022 in the United States per 100 000 population, stratified by ICD-10 coding **^a^**

| Code | Year | Deaths | Population | Crude Rate | 95% LCI | 95% UCI | Standard Error | Age Adjusted Rate | 95% LCI | 95% UCI | Standard Error | % of Total Deaths |
| --- | --- | --- | --- | --- | --- | --- | --- | --- | --- | --- | --- | --- |
| K760 | 1999 | 365 | 180408769 | 0.2 | 0.2 | 0.2 | 0 | 0.2 | 0.2 | 0.3 | 0 | 1.20% |
| K760 | 2000 | 349 | 181984640 | 0.2 | 0.2 | 0.2 | 0 | 0.2 | 0.2 | 0.2 | 0 | 1.20% |
| K760 | 2001 | 339 | 184305128 | 0.2 | 0.2 | 0.2 | 0 | 0.2 | 0.2 | 0.2 | 0 | 1.20% |
| K760 | 2002 | 409 | 186208028 | 0.2 | 0.2 | 0.2 | 0 | 0.2 | 0.2 | 0.2 | 0 | 1.40% |
| K760 | 2003 | 457 | 188090429 | 0.2 | 0.2 | 0.3 | 0 | 0.2 | 0.2 | 0.2 | 0 | 1.60% |
| K760 | 2004 | 424 | 190205384 | 0.2 | 0.2 | 0.2 | 0 | 0.2 | 0.2 | 0.2 | 0 | 1.40% |
| K760 | 2005 | 526 | 192551384 | 0.3 | 0.2 | 0.3 | 0 | 0.3 | 0.3 | 0.3 | 0 | 1.80% |
| K760 | 2006 | 523 | 195019359 | 0.3 | 0.2 | 0.3 | 0 | 0.3 | 0.2 | 0.3 | 0 | 1.80% |
| K760 | 2007 | 633 | 197403777 | 0.3 | 0.3 | 0.3 | 0 | 0.3 | 0.3 | 0.4 | 0 | 2.20% |
| K760 | 2008 | 781 | 199795090 | 0.4 | 0.4 | 0.4 | 0 | 0.4 | 0.4 | 0.4 | 0 | 2.70% |
| K760 | 2009 | 858 | 202107016 | 0.4 | 0.4 | 0.5 | 0 | 0.4 | 0.4 | 0.4 | 0 | 2.90% |
| K760 | 2010 | 978 | 203891983 | 0.5 | 0.4 | 0.5 | 0 | 0.4 | 0.4 | 0.5 | 0 | 3.30% |
| K760 | 2011 | 1108 | 206592936 | 0.5 | 0.5 | 0.6 | 0 | 0.5 | 0.5 | 0.5 | 0 | 3.80% |
| K760 | 2012 | 1232 | 208826037 | 0.6 | 0.6 | 0.6 | 0 | 0.6 | 0.5 | 0.6 | 0 | 4.20% |
| K760 | 2013 | 1371 | 211085314 | 0.6 | 0.6 | 0.7 | 0 | 0.6 | 0.6 | 0.6 | 0 | 4.70% |
| K760 | 2014 | 1661 | 213809280 | 0.8 | 0.7 | 0.8 | 0 | 0.7 | 0.7 | 0.7 | 0 | 5.70% |
| K760 | 2015 | 2008 | 216553817 | 0.9 | 0.9 | 1 | 0 | 0.8 | 0.8 | 0.9 | 0 | 6.80% |
| K760 | 2016 | 2364 | 218641417 | 1.1 | 1 | 1.1 | 0 | 0.9 | 0.9 | 1 | 0 | 8.10% |
| K760 | 2017 | 2629 | 221447331 | 1.2 | 1.1 | 1.2 | 0 | 1 | 1 | 1.1 | 0 | 9.00% |
| K760 | 2018 | 3038 | 223311190 | 1.4 | 1.3 | 1.4 | 0 | 1.2 | 1.1 | 1.2 | 0 | 10.40% |
| K760 | 2019 | 3460 | 224981167 | 1.5 | 1.5 | 1.6 | 0 | 1.3 | 1.2 | 1.3 | 0 | 11.80% |
| K760 | 2020 | 3836 | 226635013 | 1.7 | 1.6 | 1.7 | 0 | 1.4 | 1.4 | 1.4 | 0 | 13.10% |
| K760 | 2021 | 4273 | 228238412 | 1.9 | 1.8 | 1.9 | 0 | 1.6 | 1.5 | 1.6 | 0 | 20.90% |
| K760 | 2022 | 4446 | 228238412 | 1.9 | 1.9 | 2 | 0 | 1.6 | 1.6 | 1.7 | 0 | 21.70% |
| K758 | 1999 | 20 | 180408769 | 0 | 0 | 0 | 0 | 0 | 0 | 0 | 0 | 6.40% |
| K758 | 2000 | 18 | 181984640 | Unreliable | 0 | 0 | 0 | Unreliable | 0 | 0 | 0 | 5.70% |
| K758 | 2001 | 24 | 184305128 | 0 | 0 | 0 | 0 | 0 | 0 | 0 | 0 | 7.60% |
| K758 | 2002 | 19 | 186208028 | Unreliable | 0 | 0 | 0 | Unreliable | 0 | 0 | 0 | 6.10% |
| K758 | 2003 | 12 | 188090429 | Unreliable | 0 | 0 | 0 | Unreliable | 0 | 0 | 0 | 3.80% |
| K758 | 2004 | 12 | 190205384 | Unreliable | 0 | 0 | 0 | Unreliable | 0 | 0 | 0 | 3.80% |
| K758 | 2005 | 11 | 192551384 | Unreliable | 0 | 0 | 0 | Unreliable | 0 | 0 | 0 | 3.50% |
| K758 | 2006 | 11 | 195019359 | Unreliable | 0 | 0 | 0 | Unreliable | 0 | 0 | 0 | 3.50% |
| K758 | 2008 | 14 | 199795090 | Unreliable | 0 | 0 | 0 | Unreliable | 0 | 0 | 0 | 4.50% |
| K758 | 2009 | 13 | 202107016 | Unreliable | 0 | 0 | 0 | Unreliable | 0 | 0 | 0 | 4.10% |
| K758 | 2010 | 10 | 203891983 | Unreliable | 0 | 0 | 0 | Unreliable | 0 | 0 | 0 | 3.20% |
| K758 | 2011 | 11 | 206592936 | Unreliable | 0 | 0 | 0 | Unreliable | 0 | 0 | 0 | 3.50% |
| K758 | 2012 | 10 | 208826037 | Unreliable | 0 | 0 | 0 | Unreliable | 0 | 0 | 0 | 3.20% |
| K758 | 2014 | 12 | 213809280 | Unreliable | 0 | 0 | 0 | Unreliable | 0 | 0 | 0 | 3.80% |
| K758 | 2015 | 10 | 216553817 | Unreliable | 0 | 0 | 0 | Unreliable | 0 | 0 | 0 | 3.20% |
| K758 | 2016 | 14 | 218641417 | Unreliable | 0 | 0 | 0 | Unreliable | 0 | 0 | 0 | 4.50% |
| K758 | 2017 | 17 | 221447331 | Unreliable | 0 | 0 | 0 | Unreliable | 0 | 0 | 0 | 5.40% |
| K758 | 2018 | 12 | 223311190 | Unreliable | 0 | 0 | 0 | Unreliable | 0 | 0 | 0 | 3.80% |
| K758 | 2019 | 29 | 224981167 | 0 | 0 | 0 | 0 | 0 | 0 | 0 | 0 | 9.20% |
| K758 | 2020 | 19 | 226635013 | Unreliable | 0 | 0 | 0 | Unreliable | 0 | 0 | 0 | 6.10% |
| K758 | 2021 | 15 | 228238412 | Unreliable | 0 | 0 | 0 | Unreliable | 0 | 0 | 0 | 7.40% |
| K758 | 2022 | 95 | 228238412 | 0 | 0 | 0.1 | 0 | 0 | 0 | 0 | 0 | 46.80% |

**^a^** Query Date: May 13, 2023 2:26:33 PM
